# Gaze, behavioral, and clinical data for phantom limbs after hand amputation from 15 amputees and 29 controls

**DOI:** 10.1038/s41597-020-0402-1

**Published:** 2020-02-20

**Authors:** Gianluca Saetta, Matteo Cognolato, Manfredo Atzori, Diego Faccio, Katia Giacomino, Anne-Gabrielle Mittaz Hager, Cesare Tiengo, Franco Bassetto, Henning Müller, Peter Brugger

**Affiliations:** 1 Department of Neurology, University Hospital of Zurich, Zurich, Switzerland; 2 Department of Psychiatry, Psychotherapy and Psychosomatics, Zurich Psychiatric University Hospital, Zurich, Switzerland; 3 Information Systems Institute, University of Applied Sciences Western, Switzerland (HES-SO), Sierre, Switzerland; 4 Rehabilitation Engineering Laboratory, Department of Health Sciences and Technology, ETH Zurich, Zurich, Switzerland; 5 Clinic of Plastic Surgery, Padova University Hospital, Padova, Italy; 6 Department of Physical Therapy, University of Applied Sciences Western Switzerland (HES-SO), Leukerbad, Switzerland; 7 Medical faculty, University of Geneva, Geneva, Switzerland; 8 Neuropsychology Unit, Valens Rehabilitation Centre, Valens, Switzerland

**Keywords:** Sensorimotor processing, Human behaviour, Medical research

## Abstract

Despite recent advances in prosthetics, many upper limb amputees still use prostheses with some reluctance. They often do not feel able to incorporate the artificial hand into their bodily self. Furthermore, prosthesis fitting is not usually tailored to accommodate the characteristics of an individual’s phantom limb sensations. These are experienced by almost all persons with an acquired amputation and comprise the motor and postural properties of the lost limb. This article presents and validates a multimodal dataset including an extensive qualitative and quantitative assessment of phantom limb sensations in 15 transradial amputees, surface electromyography and accelerometry data of the forearm, and measurements of gaze behavior during exercises requiring pointing or repositioning of the forearm and the phantom hand. The data also include acquisitions from 29 able-bodied participants, matched for gender and age. Special emphasis was given to tracking the visuo-motor coupling between eye-hand/eye-phantom during these exercises.

## Abstract (summary)

**Table:** 

Measurement(s)	phantom limb sensation • muscle electrophysiology trait • first person video • body movement coordination trait • head movement trait • eye-hand coordination • completion time
Technology Type(s)	questionnaire • electromyography • eye tracking device • Accelerometer • accelerometer and gyroscope • data transformation • Foot Pedal Device
Factor Type(s)	age • sex • handedness • amputation side • amputation cause • years since amputation • residual limb length • prosthesis • painful limb pain • non-painful phantom limb sensations • body representation in dreams • referred sensations • obstacle shunning and obstacle tolerance • impact of the amputation on quality of life • motor imagery
Sample Characteristic - Organism	Homo sapiens

Machine-accessible metadata file describing the reported data: 10.6084/m9.figshare.11815263

## Background & Summary

Following hand loss, dramatic changes in an individual’s daily life can occur. Hand amputees often struggle to accomplish several everyday activities, and many of them are destined to social isolation and unemployment^1,2^. This may be partly explained by the self-reported lack of feeling and acceptance of a prosthesis as one’s own limb^3^ or of embodiment, as described by “*the ability to process information through external objects at the sensory, motor and/or affective levels in the same way as the properties of one’s own body parts”*^4^. Of crucial, yet largely neglected importance in designing high-tech prostheses is their interaction with phantom limb sensations (PLS), that is, the continuous phenomenal presence of sensory, motor and postural aspects in the missing body segment^5^.

PLS are felt by up to 95% of the amputees, with variable onset and duration^6^. They can be experienced as painful (e.g., burning, cramping, stabbing) or non-painful. Previous studies are often biased towards *painful* PLS. Wearing a functional prosthesis is generally considered to reduce pain^7^. However, with regard to non-painful PLS, some clinicians consider them a source of interference that hinders the successful incorporation of a prosthesis into the body schema^8^. Other authors rather emphasize that the vivid presence of a PLS may facilitate the dexterous use of an artificial limb^9,10^. PLS are generally localized in the extracorporeal space, beyond the visible anatomical borders of the residual limb. Prostheses and artificial limbs are physical matter that can overlap the phenomenal space of a phantom limb. When this happens, approximately 50% of amputees still feel their phantom limb – if only in superposition with physical matter. In the other 50%, the phantom limb disappears or is withdrawn within the stump^11^. We recently proposed the definition of Obstacle Tolerance (OBT) and Obstacle Shunning (OBS) for these two fundamentally different types of behavior^12^. Only a few clinical observations document this interaction between mind and matter^11,12^. However, the characterisation of this interaction appears crucial, as it might represent a key predictor of how fast and how satisfactory the embodiment of a prosthesis may take place.

This article presents independent datasets that include gaze, surface electromyography (sEMG), accelerometry as well as behavioral data from exercises conducted within the MeganePro project (Myo-Electricity, Gaze and Artificial-intelligence for Neurocognitive Examination & Prosthetics), an interdisciplinary and multicenter project which aimed at (i) improving the control of myoelectric hand prosthesis and (ii) understanding the neurocognitive alterations and clinical parameters in hand amputees.

The first aim of MeganePro is addressed in our parallel contribution presenting the MeganePro dataset 1 (MDS1). MDS1 refers to exercise 1, examining grasping movements using gaze and computer vision^13^.

The present contribution addresses the second MeganePro aim and presents MeganePro dataset 2 (MDS2)^14^, MeganePro dataset 4 (MDS4)^15^ and MeganePro dataset “Clinical Interview and Neurocognitive Tests in Amputees” (MDSInfo)^16^.

MDS2 contains the multimodal data for exercise 2, which tapped into the eye-hand coordination in able-bodied subjects and eye-phantom coordination in amputees during motor imagery (MI) and motor execution (ME) of visually-guided pointing movements. Fitts’ law^17^ describes the speed-accuracy trade -off when aiming with constant accuracy to point to an ever smaller target area: the total movement duration is inversely related to the logarithm of the target width. Such speed-accuracy effects were previously exploited for assessing the dexterous use of myoelectric prostheses. While in Bouwsema *et al*.^18^ kinematics and a few clinical measures were collected, in a more recent study^19^, visuo-motor behaviour was analysed adopting a visually-guided pointing paradigm. Here, we recorded multimodal data that can be analysed with reference to a participant’s individual experience of PLS.

MDS4 contains the multimodal data for exercises 4 and 5. These exercises systematically study OBS and OBT. The hand/phantom is repositioned from a starting to an end point in the presence of hindering physical matter, an “obstacle”, in between these points. To the authors’ knowledge, no previous literature has attempted to quantitatively characterize phenomena that are otherwise only clinically observed and described with self-report measures.

The dataset MDSInfo contains clinical and behavioral tests that are sensitive to detect multiple attributes that define a phantom limb, including OBS and OBT, and to provide scores of developments over time, intensity, frequency, vividness, and emotional connotations for each attribute. Other meaningful phenomena such as motor imagery, i.e., the capacity to evoke a movement without the overt action^20^, and the impact of the amputation condition on an individual’s quality of life are also reported. Thanks to this dataset, neurocognitive alterations and clinical parameters of PLS can be analysed in relation to the performance to all the exercises.

The analyses presented in this article validate the procedures and illustrate the usefulness of the data for a broader community of researchers in both prosthesis design and in the psychological and neuroscientific sequelae of hand amputation.

## Methods

### Participants

Fifteen transradial hand amputees (13 men, 2 women, mean age: 47.13 ± 14.16 y; mean years since amputation: 9.5 ± 8.5) and 29 able-bodied controls (26 men, 3 women, mean age: 47.31 ± 15.18) participated in the study. Out of the amputees, 47% were using a myoelectric prosthesis (mean hours/day: 10.06 ± 5), 33% were using a cosmetic prosthesis (mean hours/day: 7.2 ± 3.6), and 20% were using a body-powered one (mean hours/day: 13.3 ± 3.6). All amputees experienced non-painful PLS with high interindividual variability in terms of onset, intensity, and frequency. About 73% of the amputees reported painful PLS. Shrunk sensations were observed in 60% of them. About 86% of the amputees could freely move the phantom, while the other 13% of the amputees reported the feeling of having the phantom limb stuck in a certain position, i.e., frozen phantom^21^. This was tested by asking the participants to perform phantom finger tapping and phantom fist movements. OBS and OBT were observed in 46% and 54% of amputees. The OBS and OBT groups did not statistically differ in age (p = 0.37) nor years since amputation (p = 0.23) as tested by a two-samples t-test. Of all fifteen participants, eight dreamed about themselves as able-bodied, two as amputated, and a mixed pattern was observed in one amputee. Two amputees were not able to remember their dreams. One amputee could not recall the dreams vividly. Body representation in dreams is not available for one participant because of the language barrier during data collection. Referred sensations were reported in 40% of the amputees. Table 1 displays an overview of the demographical and clinical parameters presented above. For the information relative to able-bodied controls, we invite the reader to see Table 1 in Cognolato *et al*.^13^.

**Table 1:** Overview of amputees’ clinical and demographical characteristics.

ID	Sex	Age	Most used prosthesis	Use of prosthesis (Hours/Day)	Painful PLS	Non painful PLS	Shrunk	Frozen Phantom	Ostacle Shunning or Tolerance	Body Representation in Dreams	Referred Sensation
101	Male	52	Cosmetic	8	YES	YES	YES	NO	OBT	Intact limb	YES
102	Male	39	Cosmetic	9	YES	YES	YES	NO	OBS	Mixed	NO
103	Male	63	Myo-open-close	15	YES	YES	YES	NO	OBS	No dreams at all	NO
104	Male	49	Myo-open-close	9	YES	YES	YES	NO	OBT	Intact limb	NO
105	Male	73	Body-powered	16	YES	YES	YES	YES	OBT	Not vivid dream recall	NO
106	Male	70	Body-powered	8	YES	YES	NO	NO	OBT	Intact limb	NO
107	Male	36	Body-powered	16	YES	YES	YES	NO	OBT	Intact limb	NO
108	Male	35	Myo-open-close	16	YES	YES	YES	NO	OBS	No dreams at all	NO
109	Male	65	Cosmetic	8	YES	YES	NO	NO	OBS	Intact limb	YES
110	Male	38	Myo-open-close	9	YES	YES	NO	NO	OBS	Intact limb	NO
111	Male	38	Myo-open-close	5	NO	YES	YES	YES	OBT	Language barrer	YES
112	Female	33	Cosmetic	10	NO	YES	YES	NO	OBT	Amputated limb	NO
113	Male	28	Myo-open-close	4.5	NO	YES	NO	NO	OBS	Amputated limb	YES
114	Male	52	MYo-open-close	16.5	NO	YES	NO	NO	OBT	Intact limb	YES
115	Female	36	Cosmetic	1	YES	YES	NO	NO	OBS	Intact limb	YES

### Ethical requirements

Participants were provided with a written and oral explanation of the procedure and gave the signed informed consent form as a first step. One of the participants expressed his consent to publish identifiable images by filling in a form.

The experimental protocol, configured as a multi-center study, complies with the principles expressed in the Declaration of Helsinki (1964) and it was approved by the Ethics Commission of the Province of Padova in Italy (NRC AOP1010, CESC 4078/AO/17) and the Ethics Commission of the Canton of Valais in Switzerland (CCVEM 010/11).

### Acquisition setup

#### MDS2 and MDS4

The acquisition setup designed for exercises 2, 4 and 5 included acquisition hardware and software specifically developed for recording gaze, video in the participant’s first-person perspective, sEMG, accelerometer as well as behavioral data from multiple devices. The acquisition software included a backend, a media player, a text-to-speech engine, and a graphical user interface. The backend was developed in C++, and its primary objective was to acquire the data from the different devices and store them in the laptop with as low latency and the number of packets lost as possible. Furthermore, the backend applies a high-precision timestamp to the recorded data. These timestamps allow synchronizing the modalities during the post-processing step.

Vocal instructions synthesized by the text-to-speech engine guided the participant through the trails of the exercise. This solution allowed us to maintain a high quality testing environment, as it does not introduce any visual distractions that may have biased the users’ gaze behavior. In addition, the instructions were prepared in Italian, English, French, and German, covering the languages spoken by all the participants. A graphical user interface was also included and allowed the experimenter to handily interact with the software and conduct the experiment.

Gaze and first-person perspective videos were collected with the Tobii Pro Glasses 2 (Tobii AB, Sweden, http://www.tobiipro.com/). This device is a lightweight, portable, and unobtrusive eye tracker equipped with a Full HD camera, an Inertial Motion Unit (IMU), and a microphone. The Tobii Pro glasses (Tobii AB, Sweden) can be worn in the same manner as standard glasses and various nose pads can be chosen to guarantee the best tracking and comfort. Furthermore, corrective lenses can be applied in case of users with a visual impairment. The device includes a recording unit to which the glasses are connected. The recording unit provides the power supply through a rechargeable battery, stores the data onto a SD card, and can communicate wirelessly with other devices (e.g., a personal computer). The Tobii Pro glasses (Tobii AB, Sweden) allowed measuiring of the gaze position with respect to the first-person perspective video recorded by the scene camera. The gaze point was then overlapped onto the scene camera video locating where participants were looking. The Tobii Pro glasses (Tobii AB, Sweden) also returned an estimation of the gaze position in three-dimensional coordinates, the position, and diameter of the pupils, as well as the acceleration and the angular velocity of the participant’s head.

Muscle electrical activity and inertial data were recorded with a Delsys Trigno Wireless sEMG system (Delsys Inc., USA, http://www.delsys.com/). This device is equipped with 16 electrodes that communicate wirelessly with a base station. Four silver bar contacts allow each electrode to record the EMG signal at the skin level. In addition, a triaxial accelerometer is embedded in each electrode. The base unit receives the data streamed by the electrodes with a sampling rate of 1926 Hz for the EMG and 148 Hz for the accelerometer. We set the accelerometer range to ±1.5 g. For this range, a noise of 0.007 g (RMS), and an offset error of ±0.201 g for the X and Y axes and 0.201 g to −0.343 g for the z-axis are reported^22^. These data can then be accessed via a personal computer connected to the base station. For a more detailed description of the acquisition software and most of the devices used in the present study, see Cognolato *et al*.^13^.

Moreover, a footswitch was employed to collect the time to completion of a trial. The footswitch was connected to the acquisition laptop via USB and used as an additional keyboard. Thus, participants were able to autonomously input events into the acquisition software by pressing the footswitch. This solution let the participant free to focus on the execution of the exercise and fostered a natural upper limb movement. A footswitch was preferred over manually provided keypresses as it ensures the absence of any contamination of the MI and ME of the upper limbs by a motor response with the same effector, as they did, for example, in Gallo *et al*.^23^.

**Fig. 1:**
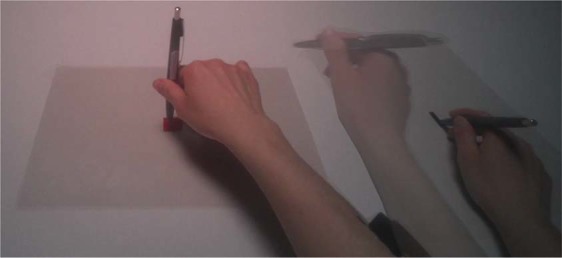
Acquisition setup for exercise 2 and the movement performed to point the target with the tip of the pen. The red square in the middle of the scene and the black rectangle on the right of the scene represent the pointing and delimiting targets, respectively.

The acquisition setup specifically for exercise 2 (MDS2) consists of five target squares, referred to as *pointing targets*, and a rectangular target. The pointing targets consisted of red squares of various sizes printed on the center of transparent sheets. They served as target for the (imagery) pointing movements to be performed with the tip of a pen. The sides of the red squares were 1.25 mm, 2.5 mm, 5 mm, 10 mm, and 20 mm, in line with a previous study^24^. The delimiting target, indicating the starting point of the movement, was a black rectangle printed on a transparent plastic sheet. The center of the smallest pointing target was aligned to the participant’s body-midline and was fixed at a distance of 47 cm from the border of the table. The starting point of the (imagery) movements, marked with the delimiting target, was horizontally aligned with the pointing target at a distance of 32.6 cm (see Fig. 1).

**Fig. 2:**
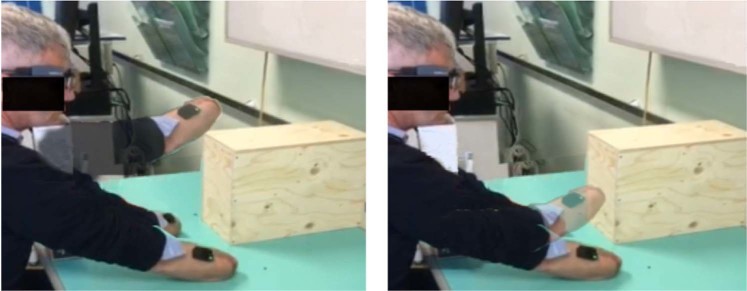
Acquisition setup of exercises 4–5 and the sequence of movements performed to reposition the (phantom) hand. The start and end points were delimited by black squares. (**a**) Exercise 4: The participants are required to move the hand/stump around and above the obstacle. (**b**) Exercise 5: The amputees are asked to move the stump in a way that the phantom would pass through the obstacle.

The acquisition setup specific for exercises 4 and 5 (MDS4) consists of an obstacle and two delimiting targets. The obstacle was a wooden rectangle box of dimensions (H × W × L) 26 × 16.5 × 31.7 cm. This item was aligned with the participant body’s midline at a distance from the border of the desk that was considered comfortable by the participant. The two delimiting targets indicated where the movement had to begin and finish. These targets were placed at 13 cm from both the left and the right side of the obstacle (see Fig. 2).

#### MDSInfo

Demographic and clinical information such as sex, age, weight, height, body mass index, laterality of amputated hand, the reason of amputation, years since amputation, type of used prosthesis specifying the hours per day and the most used prosthesis at the time to data acquisition are provided. All amputees underwent the clinical interview and cognitive tests in Italian according to their own and the examiners’ mother tongue, except in one case described in the usage notes.

The tests were administered by trained neuropsychologists and incorporate published and well-established structured tests and interviews. An extended table reporting the scores of each item of the interviews and the instructions for decoding the scores can be found in MeganePro Participants information Dataset (MDSinfo)^16^.

##### Handedness

The amputees’ hand preference for skilled activities prior to amputation was assessed by means of the FLANDERS questionnaire^25^, which lists 10 skilled activities and provides a score which indicates left, right, or ambidextrous hand preference.

##### Painful and non-painful phantom limb sensations

The “*Phantom and Stump Phenomena Interview*”^26^ *(PSPI)* was administered. It starts with the characterization of painful PLS and checks the presence of 18 pain descriptors (e.g., the pain is burning, grueling, or exhausting), that partly overlap with those presented in the McGill Pain Questionnaire-2^27,28^. For the specification of the pain descriptors and all the single items, we invite the interested reader to inspect the extended table in the MDSinfo^16^.

Detailed reports are presented on the intensity and frequency of painful PLS (computed as the ration intensity/frequency, according to Lyu *et al*.^29^). Duration of these PLS was protocolled as well and the type (and effectiveness) of treatment, if applicable. The strategies a patient may have adopted to reduce pain and the extent to which the patient could benefit from them were also inquired, as well as environmental and emotional factors that can increase or decrease the pain. The same detailed questions were also asked with respect to non-painful PLS and residual limb sensations. They comprise, among others, length, girth, position in space, and changes over time, spontaneous vs. intentional movement of the phantom hand and temperature sensations. For each attribute, scores of intensity, frequency, and magnitude are given in the dataset. Shrinkage and elongation of the phantom are also reported in terms of onset, intensity, frequency, and changes over time.

##### Body representation in dreams

The PSPI also includes a section on body representation in dreams. The way an amputee represents him/herself in dreams have been taken as an implicit index of distress during the waking state and was found to be associated with specific aspects of PLS^22,30,31^.

##### Referred sensations

The PSPI was slightly extended to describe presence and intensity of referred sensations, that are, sensations localized to the phantom hand after a tactile stimulation of a remote zone of the amputee’s body (“trigger zone”). In accordance with the literature^5^, the most common trigger zone is the participant’s face. The investigation of referred sensations has proven important. Indeed previous studies showed that the successful embodiment is associated with the referral of sensation to the prosthesis^32^.

##### Obstacle shunning and obstacle tolerance

The “*Structured Interview on Phantom Sensations”*^33^ was particularly useful for the assessment of Obstacle Tolerance versus Obstacle Shunning. OBT and OBS are measured in the following way: the amputee is asked to slowly approach the stump to a wall and indicate, qualitatively and quantitatively, any changes relative to the baseline vividness of the phantom limb. Two further tests require the participant to give a similar rating, but not upon approaching a wall, but instead on approaching the examiner’s body and, finally, the amputee’s own body. A distinction between OBS and OBT in relation to the nature of the physical matter (non-biological vs. biological) seems desirable, as the different forms could be predictive of different aspects of a prosthesis (embodiment vs. use in social contexts).

##### Impact of the amputation on the quality of life

Special attention is paid to the functional impact of the amputation on the amputee’s daily life activities as assessed by the *“Disabilities of the Arm, Shoulder and Hand questionnaire”*^34^ adapted for hand amputation. Patients were asked to rate their ability to perform daily life activities on a 5-point Likert scale.

##### Motor imagery

The questionnaire “*Vividness of Movement Imagery Questionnaire-*2 *– VMIQ*2”^35^ was used to inquire a participant’s MI capabilities in different modalities, namely kinaesthetic and visual. The section on visual MI considers two different visual perspectives: (i) an external one, where participants are explicitly asked to watch themselves performing the movements from an external point of view, thus adopting a third-person perspective; (ii) an internal visual imagery condition, where a first-person perspective has to be taken. The VMIQ2 was administered to both amputees and able-bodied participants. Versions adjusted to a participant’s native mother tongue were used.

### Acquisition protocol

#### MDSInfo

After signing the informed consent form and having provided demographical data, amputees and able-bodied controls underwent the clinical interview and neurocognitive tests. During both the tests and clinical interview, amputees were not wearing their prostheses. For amputees, the order of administration of the tests was the following:

(i) “*Flanders questionnaire*”, (ii) “*Phantom and Stump phenomena Interview”*, (iii) *“Structured interview on Phantom Sensations”*, (iv) *“Disabilities of the Arm, Shoulder and Hand Questionnaire”*, (v) “*Vividness of Movement Imagery Questionnaire-2 – VMIQ2*”.

The clinical interview was conducted before the beginning of the exercises described in this manuscript but after the completion of exercise 1^13^. This allowed participants to rest between the exercises in order to prevent muscular fatigue on the residual limb.

#### MDS2 and MDS4

The exercises were conducted with the participant comfortably sitting in front of a desk. A Delsys Trigno electrode was positioned on each forearm. When possible, the sensors were placed at the level of the e*xtensor digitorum superficialis*, identified by palpation. This was not a strict requirement as in this experiment, the inertial data were considered of paramount importance, while the sEMG would serve for control analyses. The participant wore the Tobii Pro glasses (Tobii AB, Sweden) and the examiner placed the footswitch beneath the desk in a position that allowed the participant to press it with the foot comfortably. As the last step, the examiner arranged the items on the desk, as described in the Acquisition Setup section.

#### MDS2. Exercise 2: Visually-guided pointing

During this experiment, participants were told to perform pointing movements with the tip of a pen from a starting location to the target (see Fig. 1). Participants underwent a training where they were told to point with the tip of a pen from the black rectangle to the red squares with horizontal and natural movements. Afterward, they were asked to imagine pointing movements without executing them and to use a motor strategy (rather than a visual one) to solve the exercise, that is, evoking the kinaesthetic and motor information. Participants were also instructed to press the pedal at the beginning and at the end of the training session trials. Speed and accuracy were equally stressed. Able-bodied controls underwent the training using their dominant hand; amputees used both their intact and phantom hands.

The experimental MI and ME blocks consisted of 8 cycles for each of the 5 pointing target dimensions, resulting in 40 trials each. The order of the presentation of the trial was randomized. A vocal instruction invited the participant to start the trial. The start and end of a trial were indicated by the participants themselves by pressing the footswitch located beneath the table. The behavioral outcome, namely the time to completion, was defined as the interval between two pedal presses. We measured the time to completion of one single pointing movement. Amputees underwent three pseudorandomised blocks: i) MI of the phantom limb, ii) MI of the intact hand, and iii) ME of the intact hand. For the able-bodied participants, these blocks were 2: i) MI and ii) ME of the dominant hand. The MI blocks always preceded the ME ones^24^.

#### MDS4. Exercise 4: Obstacle Shunning. Hand/Phantom Limb Repositioning by Moving Above an Obstacle

In exercise 4, participants were asked to imagine or reposition a (phantom) hand from point A to B, passing *around and above* an obstacle (see Fig. 2, left panel). The most distal segment of the amputees’ residual limb was aligned horizontally with the side of the obstacle, which was aligned to the body midline. One trial consisted of three times, moving from point A to B and then back to point A. For the amputees, the paradigm included 4 pseudorandomized blocks, each comprising 8 trials: i) MI of the phantom limb, ii) ME of the phantom limb, which required to move the stump, iii) MI of the intact hand, iv) ME of the intact hand. For able-bodied participants, the exercise also comprised 4 blocks: MI and ME of each of the two hands.

#### MDS4. Exercise 5: Obstacle Shunning. Phantom Limb Repositioning by Moving Through an Obstacle

Exercise 5 was performed in only 11 out of 15 amputees because it was conceptualized only after testing the first 4 amputees. Here, behavioral, gaze, and muscular patterns were measured while the phantom progressively invades the space occupied by an obstacle. The experimental paradigm and setup are the same as in exercise 4. However, amputees were asked to reposition the phantom from a point A to a point B passing *through* the obstacle (see Fig. 2, right panel). Two blocks of 8 trials each were implemented for imagery and real execution of the phantom movements.

### Post-Processing

Throughout the exercises, the acquisition devices described previously recorded raw data to files stored on disk. We developed a processing routine in MATLAB (MathWorks, Natick, MA, USA, http://www.mathworks.com/) to perform data curation (for instance, by verifying the absence of acquisition errors), to synchronize the individual modalities and finally to store them in a single unified data file. This section briefly outlines the processing procedure; for a more detailed description, the reader is referred to Cognolato *et al*.^13^.

After the acquisition ended and prior to any further processing, repetitions not correctly performed were manually invalidated. The most common mistake was a delayed press of the footswitch due to a variety of reasons, which would have been hardly identifiable in post-processing.

The first step of automated processing then involved reading the acquisition files one by one and converting them in to meaningful data structures. For all modalities, we made corrections to the recorded timestamps to bring them in a shared reference time. Since sEMG and accelerometry were sampled in batches for computational reasons, their timestamps were interpolated to obtain an individual timestamp for each sample. Furthermore, the sEMG data were filtered from outliers and powerline interference and then rectified via a moving root-mean-square with a window length of 300 samples (approximately 156 ms). Separate types of information from the Tobii Pro glasses (Tobii AB, Sweden) were grouped together if it had been measured at the same time (i.e., they were assigned an identical timestamp).

We subsequently synchronized all modalities by re-sampling each of them to the sampling rate of the gaze modality. The re-sampling was implemented via linear interpolation for real-valued signals and nearest-neighbor interpolation for discrete signals. If an acquisition was interrupted and therefore consisted of more segments, then these were concatenated at the exact point that would avoid duplicated trials. As a final part of the processing pipeline, identifying information was removed from all videos.

## Data Records

The data acquired and processed according to the procedures described above were released in two datasets on Harvard Dataverse, one for exercise 2 (MDS2)^14^ and one for exercises 4 and 5 (MDS4)^15^. The data from the clinical Interview and neurocognitive tests were also stored in a dedicated dataset (MDSInfo)^16^.

### MDS2

The dataset for exercise 2 is available in the MeganePro MDS2 (MDS2)^14^. It contains two data files in MATLAB (MathWorks, Natick, MA, USA) format for each intact participant, namely the motor and imaginary parts of the exercise. The MeganePro MDS2 (MDS2)^14^ comes with the DatasetContentCRC.sfv file that reports the dataset structure and the Cyclic Redundancy Check (CRC) value of each file. The DatasetContentCRC.sfv file can be used to check the appropriateness of the downloaded data and to have an overview of the dataset structure. For amputated participants, there is an additional data file for the exercise when executed with the stump. The filename of each file clearly specifies the participant’s ID and the experimental setting. The common fields in all data files are reported in Table 2. The target field contains integers from 1 to 5 that indicate in increasing order the targets of 1.25 mm, 2.5 mm, 5 mm, 10 mm, and 20 mm. For each of the participants and experimental settings, we also release the video from the first-person perspective encoded with MPEG-4 AVC in an MP4 container^13^.

**Table 2:** Common fields in all the data files and specific stimulus fields for exercise 2.

	Field	Dim.	Units	Description
All Exercises	acc	6	g	3-axis acceleration of the 2 electrodes
	emg	2	V	myoelectric activity of the 2 electrodes
	pedal	1		indicator for the pedal presses
	gazepoint	2		2D gaze point relative to the scene image
	gazepoint_invalid	1		invalidity indicator for “gazepoint”
	gazepoint3D	3	mm	3D gaze point in world coordinates
	gazepoint3D_invalid	1		invalidity indicator for “gazepoint3D”
	gazedirectionleft	3		3D gaze direction of the left eye
	gazedirectionleft_invalid	1		invalidity indicator for “gazedirectionleft”
	gazedirectionright	3		3D gaze direction of the right eye
	gazedirectionright_invalid	1		invalidity indicator for “gazedirectionright”
	pupilcenterleft	3	mm	3D position for the pupil center of the left eye
	pupilcenterleft_invalid	1		invalidity indicator for “pupilcenterleft”
	pupilcenterright	3	mm	3D position for the pupil center of the right eye
	pupilcenterright_invalid	1		invalidity indicator for “pupilcenterright”
	pupildiameterleft	1	mm	pupil diameter of the left eye
	pupildiameterleft_invalid	1		invalidity indicator for “pupildiameterleft”
	pupildiameterright	1	mm	pupil diameter of the right eye
	pupildiameterright_invalid	1		invalidity indicator for “pupildiameterright”
	tobiiacc	3	m s^−2^	3-axis acceleration of the Tobii
	tobiiacc_invalid	1		invalidity indicator for “tobiiacc”
	tobiigyr	3	°s^−1^	3-axis angular velocity of the Tobii
	tobiigyr_invalid	1		invalidity indicator for “tobiigyr”
	tobiits	1	s	timestamp in the Tobii clock
	vts	1	s	MP4 video timestamp
	mp4videoidx	1		counter for the MP4 video
	pts	1	s	TS presentation timestamp
	tspipelineidx	1		TS pipeline ID
	tsvideoidx	1		counter for the TS video
	ts	1	s	timestamp in the computer clock
Ex2	target	1		ID of the target size
	repetition	1		repetition counter
Ex4-5	type	1		ID of the experiment type
	repetition	1		repetition counter

Additional information on the properties of the fields are:

- The field *emg* contains the sEMG signals recorded with the Delsys Trigno electrodes. It is composed of two columns, reporting the signals recorded by the electrodes on the right and left forearm or residual limb, respectively^22^.
- The field *acc* contains the accelerometer data of the Delsys Trigno electrodes. The structure is the same as in the *emg* field but with the acceleration values of the X, Y, and Z axes of the two electrodes. Due to the placement of the electrodes, the reference system of the accelerometer has the x-axis parallel to the subject’s forearm, the z-axis orthogonal to the subject’s forearm, and the y-axis tangent to the forearm circumference.
- The gaze point position relative to the video-frame is reported in the *gazepoint* field. The position is expressed in (x, y) coordinates and their center is located on the top left corner of the video-frame^36^.
- The fields *gazepoint3D, pupilcenterleft, pupilcenterright, gazedirectionleft, and gazedirectionright* are reported in (x, y, z) coordinates. The coordinates are relative to the reference system of the scene camera, which has its origin in the center of the camera, the X axis points to the subject’s left, the Y axis points upwards, and the direction of the Z axis follows the right hand rule. The unit vectors *gazedirectionleft* and *gazediretionright* have origin in the left and right pupil centers, respectively^36^.
- *The tobiigyr* and *tobiiacc* fields contain the angular velocity and the acceleration of the Tobii glasses in the X, Y and Z axes^36^.

### MDS4

The data for exercises 4 and 5 are available in the MeganePro MDS4 (MDS4)^15^ and similarly structured. For each participant, a MATLAB (MathWorks, Natick, MA, USA) data file and the corresponding MP4 video registration file is available. For those participants who also performed exercise 5, an additional data file and MP4 video that is marked as ex5 are available. The fields in these data files are shown in Table 2, whereas the interpretation of the type identifier field is instead given in Table 3.

**Table 3:** Interpretation of the type identifier for exercises 4–5.

	ID	Description
Ex4	1	Imagined Right
	2	Motor Right
	3	Imagined Left
	4	Motor Left
Ex5	5	Imagined Stump
	6	Motor Stump

### MDSInfo

The questionnaire is published as part of the data of exercise 2 in MeganePro Participants information Dataset (MDSinfo)^16^. This file is published as a Microsoft Excel spreadsheet in the “xlsx” format. The first worksheet in this file contains all responses from all participants, with participants organized on rows and their responses in columns. The interpretation of each column is detailed in a second worksheet.

## Technical Validation

### MDS2 and MDS4

#### Error validation of gaze data

We added a calibration assessment at the beginning and end of nearly all the exercises to validate the quality of eye-tracking. Overall, we found an accuracy and precision of −0.8 +/− 25.8 pixels and −9.9 +/− 33.6 pixels on the horizontal and vertical axes, respectively. These values correspond to a real-world precision and accuracy of approximately −0.4 +/− 11.5 mm and −4.4 +/− 14.9 mm at a distance of 0.8 m. For a more in-depth description of these values, the interested reader is referred to the work in Cognolato *et al*.^13^.

#### Correspondence between motor imagery and motor execution

**Fig. 3:**
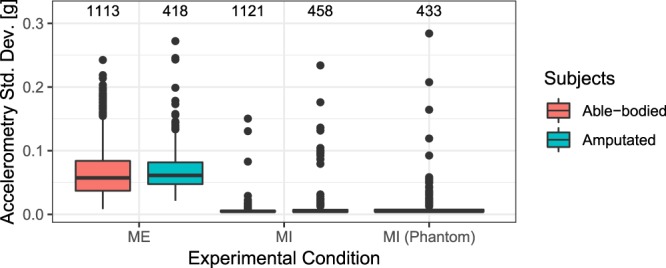
Standard deviation of the acceleration magnitude of the arm in exercise 2 for motor imagery (MI) and motor execution (ME) of pointing movements. Able-bodied individuals used the dominant hand for both MI and ME conditions. Amputees used the intact hand for both MI and ME conditions and the phantom limb for the MI condition. Medians and interquartile ranges (IQR) are plotted. Black dots represent the outliers, defined as values more than 1.5 IQR from the nearest quartile. The number of observations for each condition is indicated on the top of each column.

**Fig. 4:**
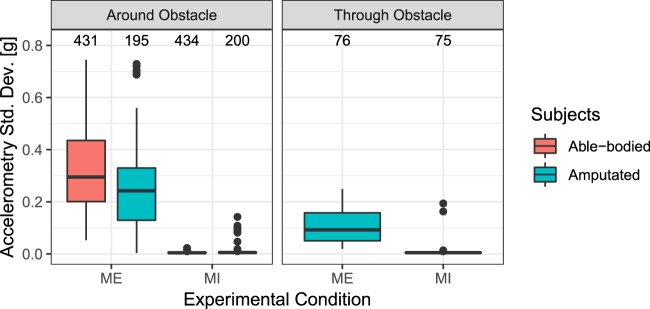
Standard Deviation of the acceleration magnitude of the arm for motor imagery (MI) and motor execution (ME) for exercises 4 and 5. In exercise 4 (left panel), all the participants moved the hands or the phantom around and above the obstacle and, in exercise 5 (right panel), amputees moved the phantom through the obstacle. Medians and interquartile ranges (IQR) are plotted. Black dots represent the outliers, defined as values more than 1.5 IQR from the nearest quartile. The number of observations for each condition is indicated on the top of each column.

The first analysis aimed at ensuring for all the exercises that participants were not moving the limb and/or the stump in the MI as compared to the ME condition. For this, we used the measurements of the accelerometers on each arm. We calculated the magnitude of acceleration for each sample. This magnitude varies with (translational) changes in acceleration of the arm, whereas it measures a constant value if the arm is static (i.e., only the constant gravity acceleration). The standard deviation of this signal within an experimental block thus indicates the amount of movement. The median movement measure is generally lower in MI than in ME both in amputated and able-bodied groups and for all the exercises (see Fig. 3 for the exercise 2 and Fig. 4 for exercises 4–5), confirming that MI and ME were correctly performed.

**Fig. 5:**
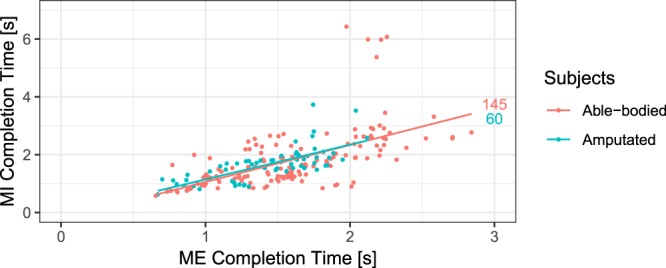
Correlation between the time to completion for motor imagery (MI) and motor execution (ME) conditions in exercise 2 in able-bodied (in red) and amputated (blue) groups. The number of observations for each condition is indicated at the end of each line.

**Fig. 6:**
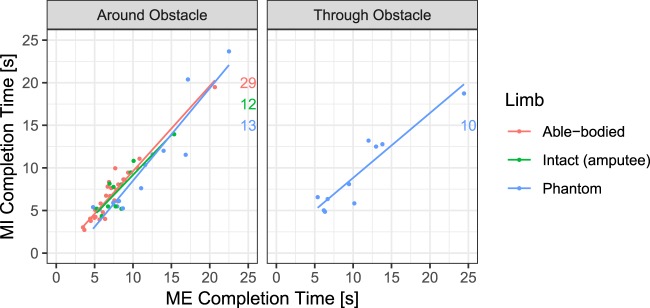
Correlation between time to completion for the motor imagery (MI) and motor execution (ME) conditions for exercises 4 and 5. In exercise 4 (left panel), all the participants moved the hands or the phantom around and above the obstacle, and in exercise 5 (right panel), amputees moved the phantom through the obstacle. The number of observations for each condition is indicated at the end of each line.

Additionally, we inspected whether the time to completion of the trial in MI and in the ME condition was correlated. The aim was to provide further evidence that in MI, participants were solving the exercise by recruiting a motor strategy. The isochronism between the time to completion of MI and that of ME was previously taken as evidence of the involvement of motor representation in MI (e.g., Parsons, 1994)^37^. As illustrated in Fig. 5, the correlation between the time to completion of MI and ME was highly significant in exercise 2 in the amputated group for the intact hand (Pearson’s correlation coefficient, r = 0.72, p = 1.309*10^−10^) and in the able-bodied control group for the dominant hand (Pearson’s correlation coefficient, r = 0.59, p = 5.461*10^−15^). This correlation was also highly significant in exercise 4 (Fig. 6, left panel) for the dominant hand in the able-bodied group (Pearson’s correlation coefficient, r = 0.96, p = 5.443*10^−16^) and for the intact hand (Pearson’s correlation coefficient, r = 0.90, p = 8.172*10^−5^) and the phantom (Pearson’s correlation coefficient, r = 0.93, 3.487*10^−6^) in the amputated group. For exercise 5 (Fig. 6, right panel), this correlation was also highly significant (Pearson’s correlation coefficient, r = 0.93, p = 9.149*10^−5^).

### MDS2

#### Assessment of Fitts’ Law in Exercise 2

**Fig. 7:**
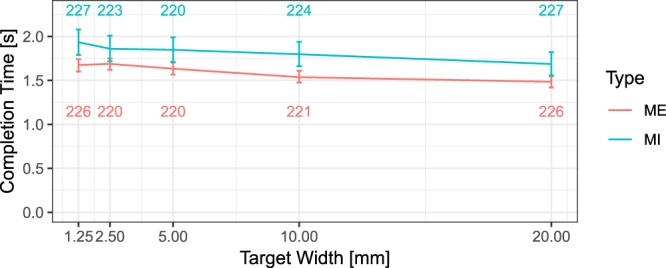
Speed-accuracy trade-off for motor imagery (MI) and motor execution (ME) in exercise 2. The number of observations for each target width is indicated on the top, for MI, and at the bottom, for ME of two lines. Mean and standard error are plotted.

In the able-bodied group, the completion time in both MI and ME was found to decrease with an increasing target width as suggested by previous studies^24^ (see Fig. 7). Indeed, the influence of the target width on the completion time in both MI and ME was taken as evidence of the involvement of common motor representation in MI and ME^24,38,39^.

Our data is also compatible with the speed-accuracy trade-off described in previous studies, according to which the difficulty of imagery and real movements is positively related to their time to completion. We further inspected whether these data obey Fitts’ law. According to this law, the index of difficulty can be expressed as a logarithmic function:

$$ID=lo{g}_{2}\frac{2D}{W}$$

where D is the target distance, and W is the target width. Fitts’ law states that the completion time (*CT*) presents a linear relationship with the index of difficulty (*ID*), as expressed in the following formula:

$$CT=a+b\ast ID$$

where *a* (intercept) and *b* (slope) are two coefficients.

In the first two analyses, one for ME and the other for MI, we averaged the time to completion of all the trials by the target width across all the 29 able-bodied participants. For ME, time to completion and index of difficulty were related according to the following positive relationship: *CT* = *1.2* + 0.053*ID, p = *0.01558, R*^*2*^ = *0.892*. For MI, the following positive relationship was found: *CT* = *1.43* + 0.055*ID, p = *0.008598, R*^*2*^ = *0.9268*. We further explored the linear relationship between the target width and the completion time, which is expressed by this negative relationship for ME (*CT* = *1.69* + (−*0.01118*2)*Target Width, R^*2*^ = *0.915, p* = *0.01075*) and this other for MI (*CT* = *1.92* + (−0.01167)*Target Width, R^2^ = *0.9447*, *p* = *0.005619*).

There is a documented modulation of movement history on Fitts’ Law^40^. We thus explored the relationship between *ID* and *CT* and of the target width and *CT* in 8 separate analyses to look at learning effects (corresponding to the 8 trials), considering the *CT* of the *n*^th^ trial for each target width averaged across the 29 able-bodied individuals. Table 4 summarises the results for ME.

**Table 4:** Linear relationship between time to completion (CT) and the index of difficulty (ID), and between CT and Target Width for the n trial.

Trial n	CT ~ ID				CT ~ Target Width			
	a	b	p value	R2	a	b	p value	R2
1	1.35	0.06	0.01	0.93	1.87	−0.01	0.04	0.81
2	1.23	0.06	0.03	0.85	1.71	−0.01	0.06	0.75
3	1.23	0.05	0.01	0.90	1.69	−0.01	0.03	0.85
4	1.20	0.05	0.03	0.85	1.66	−0.01	0.00	0.96
5	1.18	0.06	0.02	0.88	1.67	−0.01	0.01	0.94
6	1.27	0.04	0.10	0.64	1.65	−0.01	0.06	0.74
7	1.14	0.06	0.10	0.64	1.64	−0.01	0.05	0.77
8	1.27	0.04	0.05	0.77	1.58	−0.01	0.01	0.94

The results of our analyses are in line with those of other studies that show an effect of the sequence of trials on Fitts’ Law^40^. Indeed, we observed that when averaging the CT of all the trials irrespective of their sequence of appearance, compared to a nonlinear fit regression, a linear one better predicts the variability in the CT as a function of the target width. However, the opposite is true when considering the first 3 trials separately.

Overall, the data released here confirm the decreasing trend of the function. Furthermore, looking at the participants separately shows high variability in *a* and *b*. In particular, Fitts’ law holds for the performance of several subjects with variable *a* and *b* coefficients. High variability of the parameters was reported in literature^41^, although the index of performance is usually lower. The discrepancy in the magnitude of these values can be related to the points raised by Guiard & Olasfdottir^42^. Other sources of variability of the parameters can be i) the reaching time reflecting the distance between the starting and the target points and ii) the effective dimensions and the relationship between the pointer width and the target width. We hope that our data will contribute to a better understanding of the parameters that constrain the presence or absence of Fitts’ law, especially in the case of imaginary reaching movements, where precision in reaching the target cannot be measured easily.

## Usage Notes

### Repetitions and behavioral data

We invalidated the repetitions in which an error was evident during the acquisition. However, since no feedback on the correct pressing of the footswitch was given to the experimenters, other invalid events might not have been recognized by the examiners. We, therefore, consider a repetition valid if (i) it was not invalidated and (ii) if the footswitch was pressed twice, indicating the start and completion of the trial. The total number of trials that were not invalidated manually, as well as the number of repetitions containing invalid behavioral data, are summarized in Table 5. We advise the user to refer to this table when analyzing the data. We suggest the exclusion of the behavioral data of participant 109 due to the high amount of invalid repetitions.

**Table 5:** Number of invalid repetitions and number of repetitions with invalid behavioral data for all the exercises.

		Exercise 2		Exercise 4–5	
	Participant ID	Repetitions	Repetitions with invalid behavioral data	Repetitions	Repetitions with invalid behavioral data
Able-bodied Participants	11	80	7	32	1
	12	80	3	32	0
	13	80	9	32	2
	14	78	4	32	9
	15	80	4	32	5
	16	80	0	32	0
	17	78	0	32	2
	18	80	1	31	2
	19	80	1	32	0
	20	80	0	32	1
	21	80	2	32	1
	22	80	0	32	0
	23	80	0	32	0
	24	80	1	32	0
	26	80	0	32	0
	27	80	1	30	4
	28	80	0	32	3
	29	80	1	32	7
	30	78	6	32	7
	31	80	0	32	0
	32	79	3	32	1
	33	78	4	32	6
	34	80	0	31	2
	35	79	4	32	0
	36	80	7	32	4
	37	80	0	32	0
	38	80	0	32	0
	39	80	8	32	2
	40	79	9	32	0
Transradial Amputees	101	120	20	48	1
	102	120	14	32	5
	103	120	10	32	1
	104	120	16	31	3
	105	109	4	32	1
	106	118	34	40	7
	107	120	5	48	1
	108	120	2	48	0
	109	118	86	48	36
	110	120	5	44	1
	111	120	2	48	0
	112	119	3	47	6
	113	120	2	48	1
	114	n.a.	n.a.	48	0
	115	n.a.	n.a.	31	4

### Gaze

Except for the percentage of invalid data, the considerations regarding the gaze data made in Cognolato *et al*.^13^ are fully valid for this experiment as well. In brief, the acquisition of participants S111 and S114 suffered from a high number of invalid gaze data, and a specific physical condition of S115 did not allow the Tobii Pro glasses (Tobii AB, Sweden) to be perfectly stable.

### EMG

Even though of limited relevance in this study, unexpected behavior of electrode 1 was noticed for S113. The most affected exercises are exercise 4, exercise 5 and exercise 2 block executed with the residual limb. However, clipped activations were also noticed in the *imagined* and *motor* parts of exercise 2 for the same participant. The signal characteristics suggested a possible hardware issue ultimately solved by replacing the electrode.

Systematic errors and noise commonly affect the accelerometer’s data. We estimated the calibration parameters for the accelerometers embedded in the Delsys Trignlo electrodes and for the IMU of the Tobii glasses (Tobii AB, Sweden) according to the method described in Tedaldi *et al*.^43^. The calibration parameters and the original data are included in the *accelerometer_calibration.tgz* archive within the MDSScript dataset^44^.

### MDS2

Participant S013 was erroneously asked to perform exercise 2 with his non-dominant (right) hand. All other able-bodied participants performed the exercise with their dominant hand; hence, this participant’s data may not be entirely comparable to the population data.

Participant S115 did not participate in exercise 2. Furthermore, participant S114 who had both hands amputated, performed the exercise using the phantom right hand.

### MDS4

In some acquisitions, the obstacle was placed vertically on its smallest face, resulting in the height of 31.7 cm instead of 26 cm. When incorrectly positioned, the position of the box was kept identical for all conditions of exercises 4 and 5. The different positioning, associated with a height of 31.7 cm, occurred in many participants, indicated with the label “high” in Table 6.

**Table 6:** High or Low position of the obstacle in exercise 4 during each participant’s data acquisition. High: the obstacle was placed vertically on its smallest face in the height of 31.7 cm, low: this height was 26 cm.

EX4			
Box Position			
Amputees		Able-bodied	
S101	low	S011	high
S102	low	S012	high
S103	high	S013	high
S104	low	S014	low
S105	low	S015	low
S106	high	S016	high
S107	high	S017	low
S108	low	S018	low
S109	low	S019	low
S110	low	S020	low
S111	high	S021	high
S112	low	S022	low
S113	low	S023	low
S114	low	S024	low
S115	low	S026	high
		S027	low
		S028	low
		S029	low
		S030	low
		S031	low
		S032	low
		S033	high
		S034	low
		S035	high
		S036	low
		S037	low
		S038	low
		S039	low
		S040	low

### MDSInfo

Data on qualitative and quantitative aspects of PLS are generally available for all participants with only very few missing data points, as indicated in Table 7.

**Table 7:** Overview of the missing neurocognitive tests.

ID	Missing Tests	Motivation
111	VMIQ-2, DASH, Body Representation in dreams	Language Barrier
105	VMIQ-2	Time pressure
106	VMIQ-2	Participant’s wish to discontinue the experiment

### S109

Participant S109 presented a considerable number of invalid behavioral trials for all the exercises that could be explained by several factors. We suggest that the participant might not have paid attention to correctly press the pedal at the beginning and the end of the trials. We, therefore, discourage the use of participant’s S109’s behavioral data.

## Data Availability

The repository MeganePro Script Dataset^44^ also hosts the MATLAB (version 2016b, MathWorks, Natick, MA, USA) code used for the post-processing procedure and the validation scripts used to obtain the results reported in this manuscript. The README file contained in the *megane_postprocess.tgz* archive describes and reports the scripts used for the data processing. The code used for the technical validation included in this paper and to produce the corresponding figures are within the *meganepro_validation_ex245.tgz* archive. These scripts are commented in a step-by-step manner, and they can be inspected and run using R studio (Version 1.1.442). The file *ex2_validation.R* contains the code used for the technical validation of exercise 2, while the file *ex4_validation.R* was used for technical validation of exercises 4–5. The original data cannot be released to ensure the privacy of the participants. However, the provided code contains all the steps taken during each stage of the data processing and technical validation. Furthermore, they can be adapted and applied to similar tasks and similar validation/statistical questions by interested researchers.
